# Visual perception preferences in Lingnan gardens: a semantic differential survey with a convenience sample

**DOI:** 10.3389/fpsyg.2026.1750378

**Published:** 2026-03-09

**Authors:** Qiang Guo, Chun Zhu, Yile Chen, Zhao Pan, Jingwei Liang, Qingnian Deng, Liang Zheng

**Affiliations:** 1 School of Design and Innovation, Shenzhen Technology University, Pingshan, Shenzhen, China; 2 Faculty of Humanities and Arts, Macau University of Science and Technology, Tapai, Macau SAR, China; 3 School of Art and Design, Shandong Jiaotong University, Jinan, Shandong, China

**Keywords:** convenience sample, Lingnan garden, semantic difference method, subjective evaluation, visual perception preference

## Abstract

Lingnan gardens represent one of the three major schools of classical Chinese gardens. As the manifestation of regional culture and ecological wisdom in South China, they are considered irreplaceable in terms of their inherited historical context and role in shaping the spirit of locations. The aim of this study was to investigate the public’s perception of the commonalities and characteristics of Lingnan gardens as well as group differences in visual perception to accurately understand the public’s visual preferences for Lingnan gardens and promote their protection, utilization, and innovative development. A convenience sampling method was used to the semantic differential (SD) method, and Yuyin Garden in Guangzhou, Foshan Liangyuan Garden, Dongguan Keyuan Garden, and Shunde Qinghui Garden were selected as sample collection locations. A total of 120 valid data points were collected. Factor analysis, descriptive statistics and chi-square test were used to explore the perceptual dimensions and inter-group differences. The findings of the study were as follows: (1) Principal component factor analysis revealed that the four factors with the greatest impact on public visual perception were visual attention factors, visual information content factors, field of vision factors, and spatial visual factors. (2) Among the evaluation factors in the 120 questionnaires, the overall scoring trend was consistent, and the visual perception results of Lingnan gardens in different locations were similar. Natural elements received the highest evaluation score, and the public’s evaluation of this issue was the most unified. The evaluation of area size and Western cultural elements had the most significant differences, and the opinions of the respondents were difficult to unify. (3) There were two items that differed due to different groups of people and were divided into two categories: growth factors and non-growth factors, among which growth factors have a greater impact. The differences in visual perception of Lingnan gardens were explored in this study from the perspective of the public, and a quantitative translation path for analyzing the differences in visual perception of classical Chinese gardens is proposed. Although the sample size is sufficient for an initial understanding, it is recommended that future studies involve larger and more stratified samples to enhance generalizability.

## 1 Introduction

As a core carrier of regional culture and ecological esthetics, garden space not only directly reflects the public’s esthetic needs visually but also plays a crucial role in the optimization of landscape design (Gobster et al., 2007; Zhou, 2025), cultural inheritance, dissemination, and spatial evaluation through visual perception research (Gobster et al., 2007; Filova et al., 2014). With the development of garden architecture, research on preferences for mountains and water has become a key link connecting spatial design, user needs, and regional culture (Filova et al., 2014; Cai et al., 2022; Dearden, 1984). Scientific support can be provided for the refinement of garden space by analyzing the public’s visual perception patterns of garden elements.

Classical Chinese garden design is mainly divided into three major schools: Northern gardens, represented by the imperial gardens of Beijing; Jiangnan gardens, represented by the private gardens of Suzhou; and Lingnan gardens, represented by the gardens of the Lingnan region (Xu et al., 2016; Zhang, 2018). Each school has its own unique way of growing plants and arranging space that is specific to its region. Northern gardens, typified by imperial palaces, emphasize grand axes and ritual order. In these gardens, symbolic landscapes that “shrink the heavens and the earth” are often constructed through large-scale artificial hills and water features (West, 2005). Horticultural techniques highlight the ritualistic arrangement of seasonal plants and the courtly technique of stacking large stones to create artificial mountains. In Jiangnan gardens, modeled after Suzhou gardens, the freehand esthetic of “seeing the grand in the small” is pursued (Xu and Suwanthada, 2024). The spatial layout of these gardens skillfully utilizes winding waterways, interspersed corridors and bridges, and framed views to create multi-layered visual penetration (Chen, 2007; Fang, 2013; Su, 2018). The exquisite stacking of lake stones, the mimicry of flowers and trees, and the borrowing of scenery through window lattices embody horticultural techniques.

Lingnan gardens differ from the dignified, grand, and elegant style of Beijing classical gardens and from the graceful, delicate, and simple style of Jiangnan gardens (The People’s Government of Guangzhou Municipality, 2023; Zheng, 2019). Lingnan gardens are characterized by their openness, lightness, exquisite beauty, and practicality and have a history spanning over two thousand years (Zheng, 2019; Zhang W. et al., 2024). Originating in the Qin and Han dynasties, they gradually flourished through long-term development, reaching maturity in the Ming and Qing dynasties, and they continued to be developed and promoted in contemporary times (Peng, 2025; Li, 2022). Lingnan gardens achieve a high degree of unity between practicality and artistry, preserving traditional garden styles while incorporating elements from both Chinese and foreign cultures and presenting garden landscapes with distinct local characteristics and esthetic value (Zheng, 2019; Wang et al., 2025).

All three major schools of thought are part of the classical Chinese garden system. Their cultural characteristics are rooted in the imperial gardens of the north while also incorporating regional environmental features and blending together. For example, many owners of Lingnan and Jiangnan gardens had experience serving as officials in the capital, and these Cantonese officials residing in Beijing were influenced by the local trend (Tang, 2024). Furthermore, the construction of Lingnan gardens was also influenced by the prevailing cultural trends of the Jiangnan literati, who absorbed construction techniques and styles to create a “poetic” retirement home for themselves after retiring to their hometowns (Yiman and Xiaomei, 2023). The Lingnan region, being a coastal area, also served as an important window for China’s foreign trade (Korolkov, 2023), with a large influx of European culture and goods entering via the Maritime Silk Road, influencing the architectural and garden esthetics of Lingnan. These intertwined factors have given Lingnan gardens their unique style.

Current research on Lingnan gardens mainly focuses on traditional garden elements and ecological functions, such as the esthetics of door and window shapes (Wang et al., 2025), the cultural connotations of plant landscapes (Zhang J. et al., 2024), climate adaptation to hot climates (Liang and Li, 2019), decorative patterns (Zheng, 2019), courtyard space types (Xuan et al., 2023), summer heat and comfort (Xue and Xiao, 2016), three-dimensional green biomass (TGB), and environmental ecological benefits (EEBs) of Lingnan garden plants (Liping et al., 2020) and landscape narratives (Tao and Rodloytuk, 2023). However, quantitative research on public visual perception preferences for Lingnan gardens is currently relatively lacking. First, the quantitative analysis of subjective perception is insufficient. Existing studies mainly focus on the description and analysis of garden objects (such as components, plants, and decorations) and lack a systematic quantitative survey of the public’s subjective visual perception structure and preferences. Second, there is a lack of an overall perception framework. A multi-dimensional analytical framework that can integrate visual attention, content, field of view, and spatial order has not yet been established to explain how the public forms complete visual cognition in Lingnan garden space. Third, the research on group differences is scarce. There is a lack of empirical data to reveal and compare whether tourists from different demographic backgrounds (such as gender, region, age, and education level) have different visual perception patterns. Based on this, this study argues that, in the context of cultural heritage revitalization and urban renewal, understanding the public’s visual preferences for Lingnan garden spaces is crucial for accurate protection, landscape optimization, and cultural inheritance and addressing the lack of research in this field.

The semantic differential (SD) is a landscape evaluation method proposed by the psychophysical school (Ulrich, 1981). It can objectively measure the public’s intuitive perception of a landscape. First proposed by Charles E. Osgood in 1957, the SD is a quantitative research method based on psychometrics (Osgood, 1980). Its significance lies primarily in its “semantic analytical method” using language as a scale for psychological experiments. It employs semantic differentiation scales and quantitative questionnaires to score users on predetermined word pairs, obtaining quantitative analysis and research on the overall and partial aspects of the research object based on user experience. It is a method for measuring people’s psychological evaluation. This method utilizing the semantic analysis of psychological experiments represents a real-world survey approach for evaluating architectural space environments, enabling a shift from subjective to rational and quantitative analysis.

The SD method has wide applications in environmental perception, landscape assessment, and architectural space evaluation. Its applicability in the study of visual perception in Lingnan gardens demonstrates its ability to effectively capture the public’s intuitive feelings and emotional responses to garden spaces, supporting the shift from subjective description to rational quantitative analysis. This feature provided a methodological foundation for constructing a multi-dimensional visual perception evaluation framework in this study. For example, in 1996, Zhuang Weimin proposed a real-world survey method for evaluating the architectural space environment based on semantic analysis, making it possible to elevate the evaluation of architectural space from a perceptual to a rational and quantitative analysis (Zhuang, 2017). He also outlined a complete analytical survey process for environmental evaluation based on the SD method, encompassing the setting of evaluation criteria, evaluation procedures, multi-factor variable analysis methods for real-world surveys, and evaluation conclusions.

To address the identified research gaps, this study aims to systematically investigate the public’s visual perception of Lingnan garden spaces and its underlying group differences. Specifically, the objectives of this study are as follows: (1) to construct a multi-dimensional evaluation framework for the visual perception of Lingnan gardens using the semantic difference (SD) method to identify the core factors influencing public visual perception; (2) to quantify the public’s visual preferences, consensus, and points of disagreement regarding typical Lingnan garden spaces; and (3) to explore the significant differences in visual perception evaluation among different demographic groups (e.g., gender, age, education level, geographical origin) and understand their underlying patterns. To achieve these objectives, we conducted on-site SD questionnaire surveys at four representative Lingnan gardens (Yuyin Garden in Guangzhou, Foshan Liangyuan Garden, Dongguan Keyuan Garden, and Shunde Qinghui Garden), collecting valid questionnaires from 120 participants. Data analysis employed factor analysis, descriptive statistics, and chi-square test. We expect the research results to reveal the core perceptual dimensions of Lingnan gardens, quantify the consistency (or deviation) between public perception and traditional descriptions, and provide a foundational “perceptual evidence base” for the protection, design, and cultural interpretation of these heritage spaces.

## 2 Materials and methods

### 2.1 Study area: four traditional Lingnan gardens

**Figure 1:**
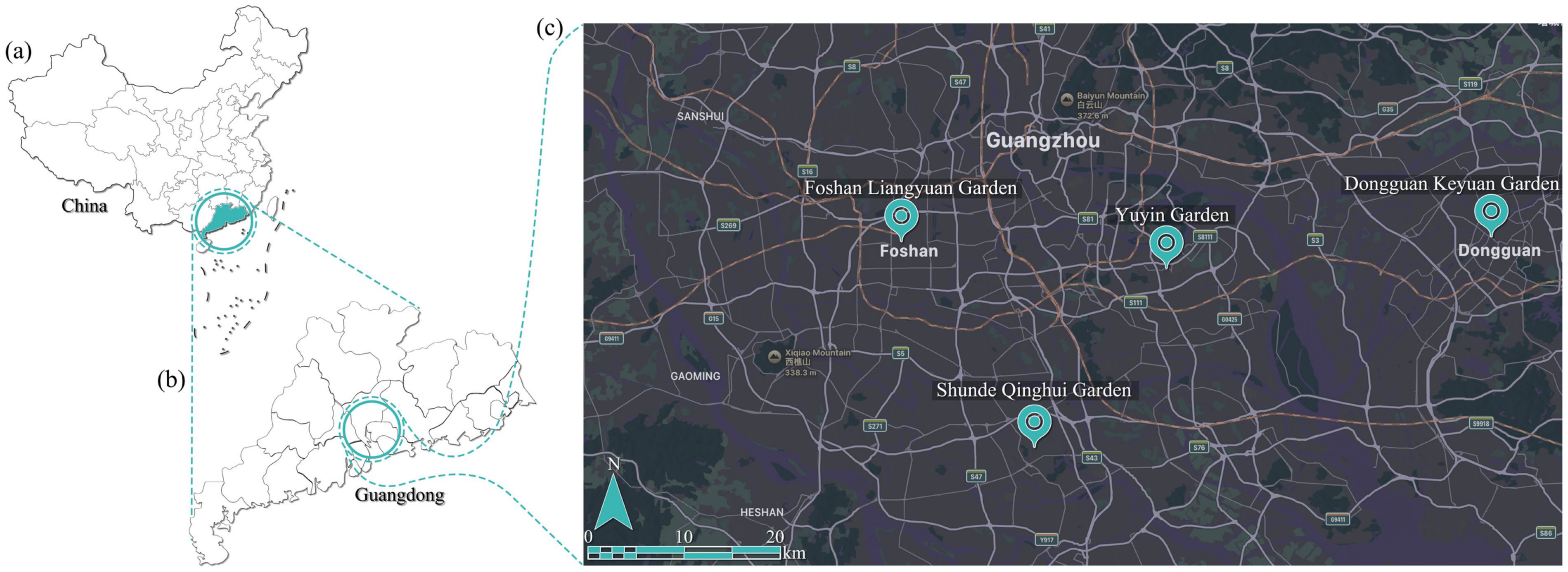
Location analysis of the study area. **(a)** Guangdong’s location in China; **(b)** the three cities where the four traditional Lingnan gardens are located; **(c)** the location of the four gardens in the Pearl River Delta.

**Figure 2:**
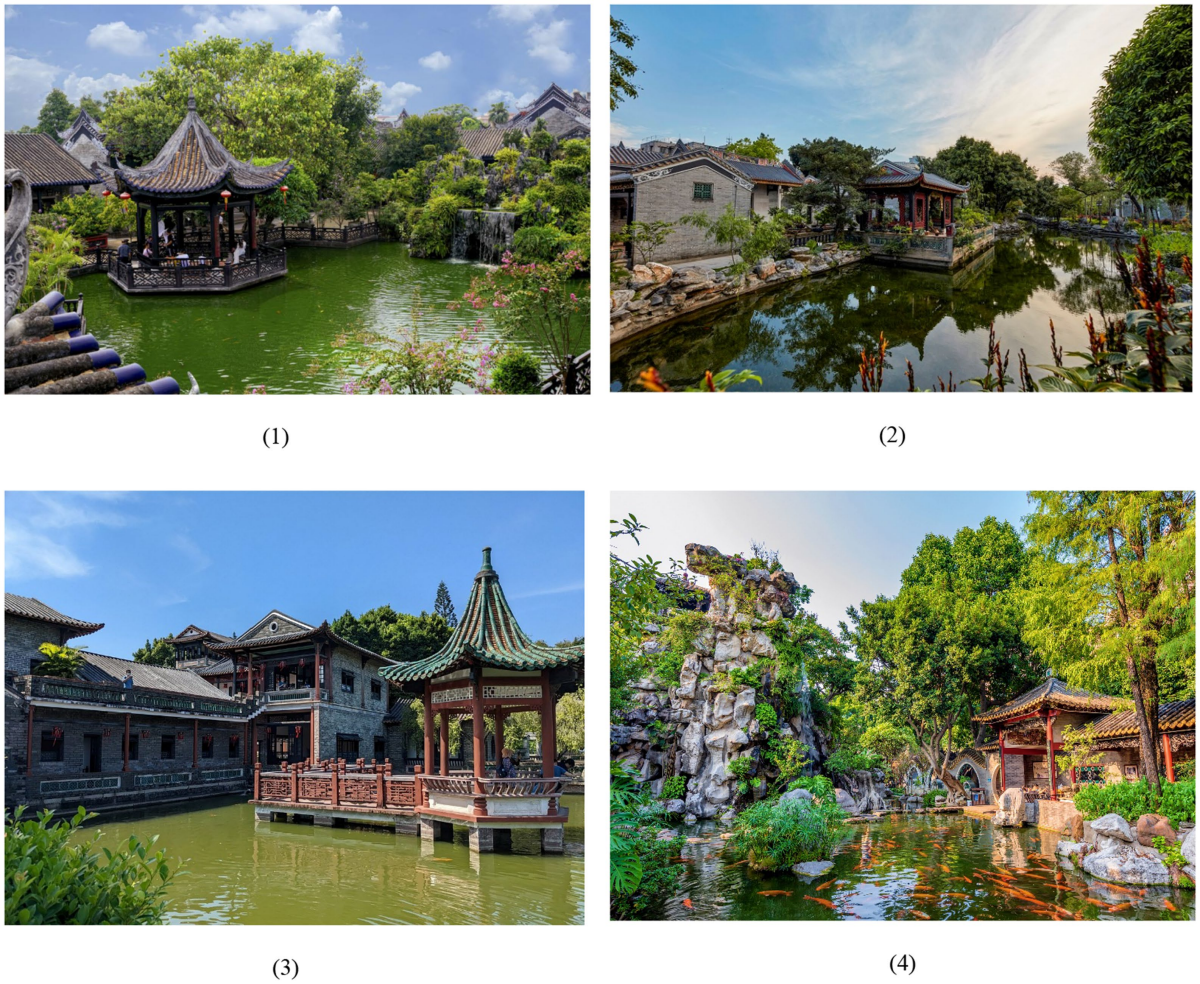
Landscape of traditional Lingnan gardens: **(1)** Yuyin Garden in Guangzhou, **(2)** Foshan Liangyuan Garden, **(3)** Dongguan Keyuan Garden, and **(4)** Shunde Qinghui Garden.

**Figure 3:**
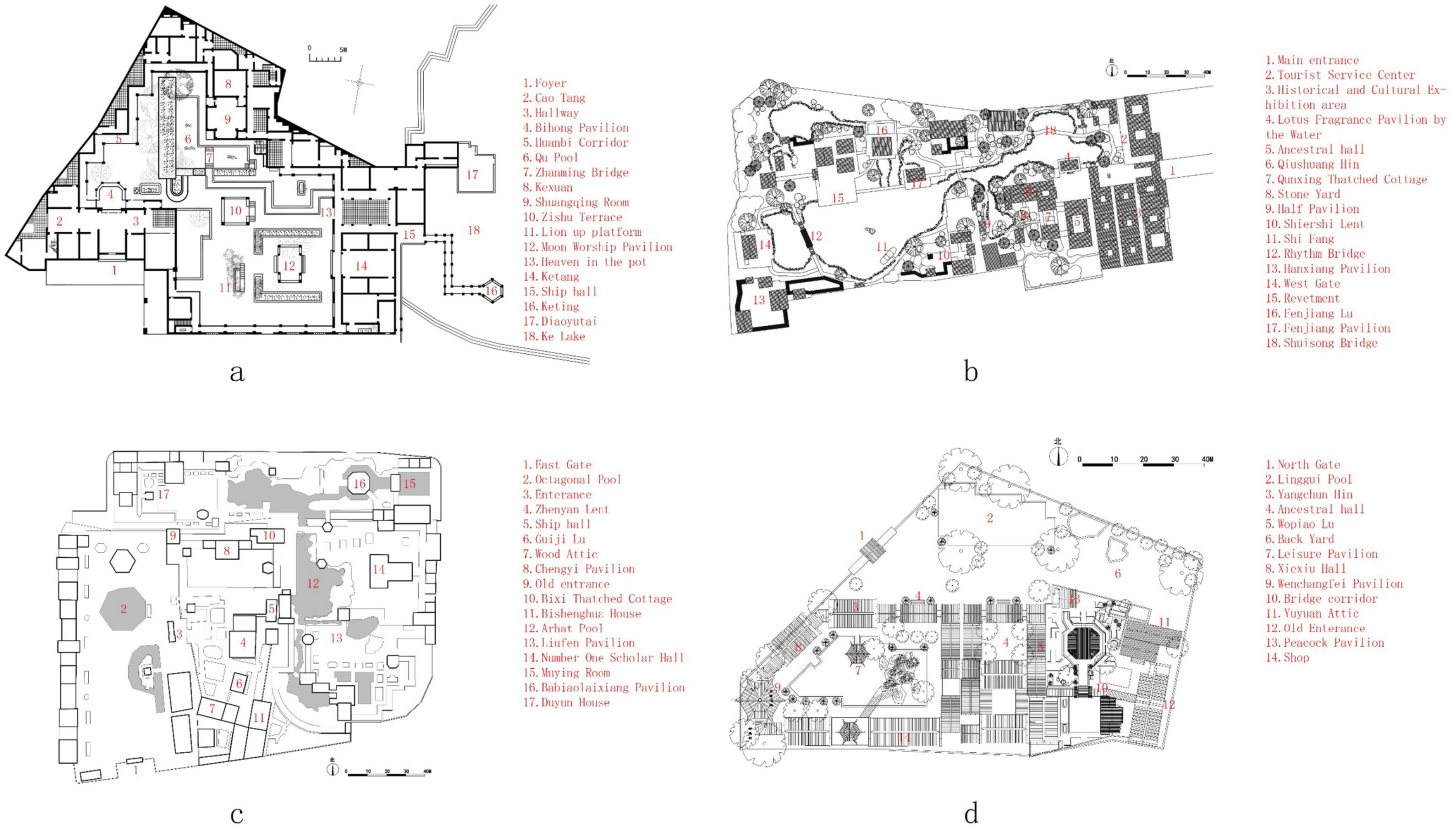
The layout of the four gardens in Lingnan: **(a)** Dongguan Keyuan Garden, **(b)** Foshan Liangyuan Garden, **(c)** Shunde Qinghui Garden, and **(d)** Yuyin Garden in Guangzhou.

Four of the most typical representatives of traditional Lingnan gardens were selected in this study: Yuyin Garden in Guangzhou, Foshan Liangyuan Garden, Dongguan Keyuan Garden, and Shunde Qinghui Garden (Zheng, 2019; The People’s Government of Guangzhou Municipality, 2024). Figure 1 shows their location. They are representative works of classical Lingnan gardens, integrating the unique geographical and cultural characteristics of the Lingnan region, such as a courtyard layout, the use of stones as mountains, and the combination of folk crafts and Western European decorative styles (Zhong, 2020; Zhang and Wang, 2021) (Figure 2). Built in 1867, Yuyin Garden was the private garden of Wu Bin, a Juren (successful candidate in the imperial provincial examination) who passed the imperial examination during the Daoguang era of the Qing Dynasty (Sihan et al., 2015). It is famous for its layout techniques of “concealing without revealing” (藏而不露) and “miniaturizing the dragon into an inch” (缩龙成寸). The rich visual layers of its architectural colors and lighting design within a limited space are highly characteristic of the Lingnan region (Figure 2a). Foshan Liangyuan Garden was built by an uncle and nephew of the Liang family during the Daoguang period of the Qing Dynasty (Figure 2b). It is characterized by its combination of residential gardens and unique rock formations (Wu, 2014; Zijian and Zhang, 2025). Dongguan Keyuan Garden was first built in 1850. It was a site of works by artists such as Ju Chao (居巢) and Ju Lian (居廉) and had a direct influence on the development of the Lingnan School of Painting. The garden’s design emphasizes seclusion and viewing. The architectural spaces are intricate and interconnected, making for fascinating exploration (Figure 2c). Shunde Qinghui Garden was originally built in the Ming Dynasty and later expanded by the Long family in the Qing Dynasty. It covers approximately 5.1 acres (0.34 hectares). The garden’s distinctive feature lies primarily in its practicality. Designed to suit the hot southern climate, it features a unique layout with sparse areas in the front and denser areas in the back and lower areas in the front and higher areas in the back (Figure 2d). However, the layout is neither empty nor cramped, and the buildings are light, flexible, open, and airy (Figure 3). The garden’s spatial arrangement uses various smaller spaces to highlight the large water garden in the courtyard. The focus of the garden’s design revolves around the water pavilion, resulting in a clear distinction between primary and secondary spaces and a well-defined structure. Selecting these four gardens as the research area serves two purposes. First, it covers the diversity of Lingnan gardens in terms of spatial types (residential gardens, suburban gardens, and courtyard gardens), different cities in the Pearl River Delta, literati interests, and local customs. Second, the high profile and visitor volume of these gardens ensure the representativeness of the research sample, providing typical and comprehensive research platforms for analyzing visual perception preferences in Lingnan garden spaces.

### 2.2 Analytical tools: semantic differential method

**Figure 4:**
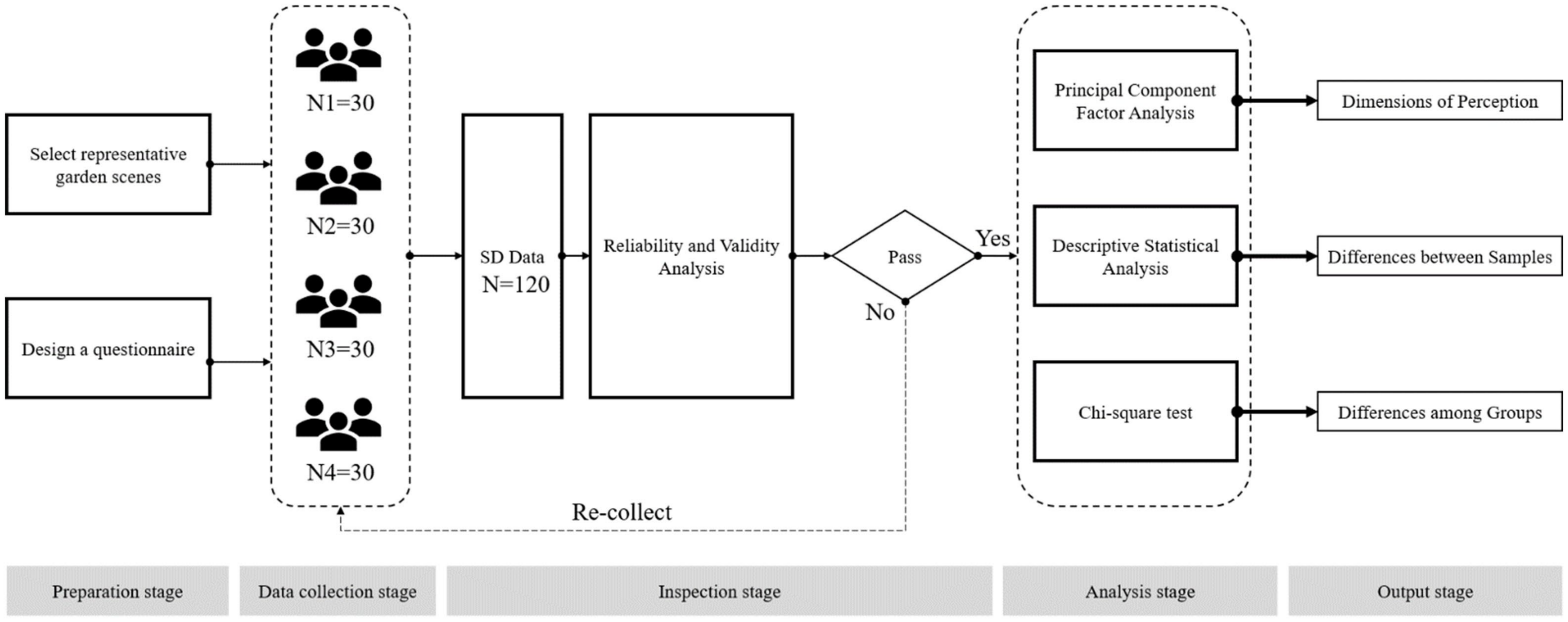
Experimental procedure framework in this study.

Architectural heritage spaces possess a unique spatial visual experience due to their distinctive construction culture and long-term historical management. We used the SD method to semantically analyze the subjective style description features involved in the Lingnan garden landscape and conduct relevant analysis based on the statistical results (Figure 4). The following steps were taken: (1) selecting participants who had just completed a tour of the gardens and soliciting their willingness to participate in the questionnaire; (2) obtaining quantitative data values through the collection of evaluation questionnaires; (3) selecting the factor scale through factor analysis; and (4) constructing the factor components of the target as data support for subsequent analysis. The four representative gardens of Lingnan were selected as the sites for the on-site questionnaire survey, in line with the study objectives.

#### 2.2.1 Evaluation criteria

The 16 SD scale term pairs in this study were obtained from five monographs on Lingnan gardens, totaling 1.316 million words (Table 1). Using these monographs on Lingnan gardens as samples, we recorded high-frequency words describing the visual characteristics of Lingnan gardens in the literature. The high-frequency descriptive words appearing in the sample are mainly: original mountain site selection (原山选址), micro-mountain and pond planning (微山规池), critical integration (临界交融), climbing high and looking far (登高望远), constructing enclosure (构筑围合), building a garden with courtyard (以庭建园), shrinking the dragon to an inch (缩龙成寸), hiding without revealing (藏而不露), filtering light to form a picture (滤光成画), and fine trees as a canopy (嘉木为盖). These high-frequency words need to be initially deconstructed or simplified for better public understanding and responsiveness. Therefore, we broke them down into 16 scale word pairs to cover as much as possible the descriptions of the visual characteristics of Lingnan gardens from the literature, ensuring that the selected items fully capture the unique visual attributes of Lingnan gardens. The evaluation criteria of the SD method consist of multiple pairs of adjectives composed of words with contrasting meanings (positive and negative). Based on spatial imagery terms such as spatial closure, transparency, and connectivity, and according to the spatial characteristics described in natural language, 16 pairs of words were selected as component factors for evaluating the visual perception characteristics of the external space of Lingnan garden architecture. The pairs of words cover three major aspects: visual cognitive law elements, routes, and the visual field. They were selected and formulated from different angles of visual cognitive law, such as the spatial impression, area size, natural elements, field of vision, elevation, degree of enclosure, visual obstruction, visual hierarchy, spatial compactness, cultural elements, visual richness, color integrity, color richness, and light and shadow perception. Among these, there is 1 overall cognitive evaluation item, 4 spatial elements, 7 visual elements, and 4 visual field elements (Table 2).

**Table 1:** Five monographs on Lingnan gardens were used to summarize the 16 terms of the SD scale.

The title of the monograph	Authors	ISBN	Total number of words in the book
Lingnan Gardens - Fujian and Taiwan Gardens (岭南园林·福建台湾园林)	Liu Tingfeng (刘庭风)	7–5,608–2,562-1	250,000
Lingnan Gardens - Guangzhou Gardens (岭南园林·广州园林)		7–5,608–2,564-8	305,000
Lingnan Gardens - Hainan, Guangxi, Hong Kong & Macau Gardens (岭南园林·海南广西香港澳门园林)		7–5,608-2563-X	270,000
Lingnan Gardens (岭南园林)	Liu Guanping (刘管平)	978–7–5,623-3837-6	251,000
Lingnan Private Gardens (岭南私家园林)	Lu Qi (陆琦)	978–7–302-32795-0	240,000

Source: Authors’ statistics.

**Table 2:** The adjective phrases are selected in the SD experiment.

No.	Evaluation objects	Adjective phrases	Category
1	Visual impression	Profound–Superficial	Overall cognitive evaluation
2	Area size	Large–Small	Spatial elements
3	Natural elements	Many–Few	Visual elements
4	View from garden entrance	Open–Limited	Visual field elements
5	View from building entrance	Open–Limited	Visual field elements
6	Satisfaction with heights	Satisfied–Lacking	Spatial elements
7	Sense of enclosure	Strong–Weak	Spatial elements
8	Visual obstruction	Many–Few	Visual field elements
9	Visual hierarchy	Rich–Simple	Visual field elements
10	Spatial compactness	Clear–Vague	Spatial elements
11	Cultural elements	Many–Few	Visual elements
12	Richness of visual content	Rich–Monotonous	Visual elements
13	Integrity of color tone	Unified–Chaotic	Visual elements
14	Color richness	Rich–Monotonous	Visual elements
15	Perception of light and shadow	Clear–Vague	Visual elements
16	Shading measures	Sufficient–Lacking	Visual elements

Source: Authors’ statistics.

#### 2.2.2 Evaluation levels

The SD scoring method can be divided into 5, 7, and 11 levels. The stages of the SD method should “avoid asymmetrical evaluation scales with 0 as the midpoint, and the rating scale should ideally be 5-7 levels”. The 7-level scale can be divided into very many, many, some, average, few, and very few, while the 5-level scale can be divided into very many, some, average, few, and very few. Whether comprising 11, 7, or 5 levels, the common characteristic of the scales is the odd number of levels. An excessive number of evaluation scale levels complicates the survey for respondents, making the survey process cumbersome and unbalanced. Conversely, an insufficient number oversimplifies the scale, making it difficult for respondents to summarize their views and thus reducing the accuracy of the evaluation results. Therefore, a 7-level scale was adopted in this study for the evaluation of 16 pairs of adjectives, with assigned values of −3, −2, −1, 0, 1, 2, and 3 symmetrically set around 0 as the central axis, facilitating subsequent statistical calculations. The 7-level rating index has adequate adaptability and can accurately reflect people’s psychological expectations. It is more precise than the 5-level rating index and clearer than the 9-level evaluation index and is less likely to produce ambiguous choices (Table 3).

**Table 3:** SD method evaluation levels.

Emotionalattitude	Extremely negative	Very negative	Relatively negative	Neutral	Relatively positive	Very positive	Extremely positive	Emotional attitude
Adjectives (antonyms)	-3	-2	-1	0	1	2	3	Adjective (positive)

Source: Authors’ statistics.

#### 2.2.3 Questionnaire design and implementation

This study conducted a questionnaire survey at four typical Lingnan gardens (Yuyin Garden in Guangzhou, Foshan Liangyuan Garden, Dongguan Keyuan Garden), employing convenience sampling. This method was chosen primarily based on the practical feasibility of the research scenario and its alignment with the research objectives. First, in public cultural heritage sites with high visitor traffic and varying visit times, it is impossible to obtain a complete visitor sampling frame for rigorous probability sampling; convenience sampling efficiently reaches visitors who have just completed their visit and have vivid memories, ensuring the timeliness and authenticity of data collection. Second, the main objective of this study is to explore the potential dimensions and group differences in public visual perception, according to discovery-based and descriptive preliminary research. Convenience sampling provides an effective way to quickly obtain preliminary empirical data for this type of research. Although this method may bias the sample towards visitors willing to stay and provide feedback within a specific time period, we took the following measures to improve the reliability and representativeness of the data: questionnaires were distributed continuously for several weeks during November 2024, covering weekdays and weekends; visitors of different ages and genders were randomly invited to participate at the garden exit areas; and a concise questionnaire design (approximately 15 min to complete, see Appentix Questionnaire Survey on the Perception of Lingnan Garden Landscape) was used to improve participation and data quality. Therefore, within the exploration framework and field constraints of this study, convenience sampling is a reasonable choice to balance research rigor and operational feasibility. Its limitations will be explained in the discussion section and will provide a basis for future confirmatory studies using stratified or random sampling.

Furthermore, this study focused on the entire Lingnan gardens. To enhance the universality of the research, the samples were collected from four representative gardens. It was hoped that this would provide a broader range of sample collection. A total of 120 valid questionnaires were collected, and no duplicate questionnaires were filled out.

**Figure 5:**
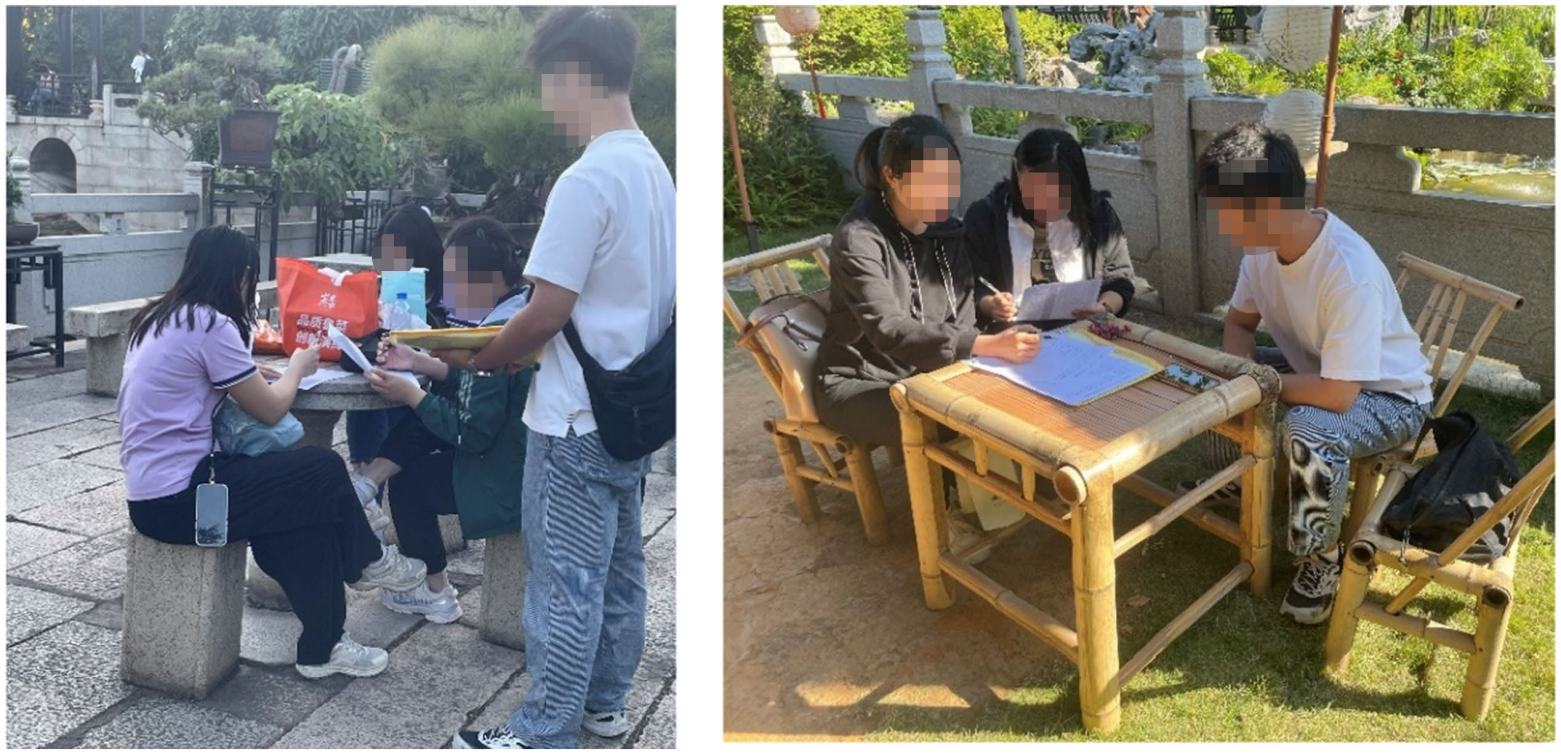
Questionnaire field survey section on-site.

A questionnaire with 16 semantic differential items and 4 demographic questions was designed. The questionnaire was distributed randomly to visitors to the gardens to ensure the authenticity and validity of respondents. In this study, 120 valid questionnaires were collected, resulting in a 100% response rate (Figure 5).

## 3 Results

### 3.1 Descriptive statistical analysis

#### 3.1.1 Sample distribution

The basic information in the questionnaire sample was obtained through coding, sorting, and statistical analysis of the collected statistical results (Table 4).

**Table 4:** Frequency distribution of basic information of questionnaire samples.

Item		Keyuan Garden		Liangyuan Garden		Qinghui Garden		Yuyin Garden		Total (4 Gardens)	
		Frequency	Percentage	Frequency	Percentage	Frequency	Percentage	Frequency	Percentage	Frequency	Percentage
Gender	Male	19	63.3%	11	36.7%	11	36.7%	13	43.3%	54	45.0%
	Female	11	36.7%	19	63.3%	19	63.3%	17	56.7%	66	55.0%
	Total	30	100.0%	30	100.0%	30	100.0%	30	100.0%	120	100.0%
From/Outside Guangdong Province	Within the province	18	60.0%	22	73.3%	18	60.0%	23	76.7%	81	67.5%
	Outside the province	12	40.0%	8	26.7%	12	40.0%	7	23.3%	39	32.5%
	Total	30	100.0%	30	100.0%	30	100.0%	30	100.0%	120	100.0%
Education level	High school and below	5	16.7%	6	20.0%	0	0%	7	23.3%	18	15.0%
	University	21	70.0%	21	70.0%	29	96.7%	21	70.0%	92	76.7%
	Graduate and above	4	13.3%	3	10.0%	1	3.3%	2	6.7%	10	8.3%
	Total	30	100.0%	30	100.0%	30	100.0%	30	100.0%	120	100.0%
Age group	16–25 years old	10	33.3%	11	36.7%	23	76.7%	5	16.7%	49	40.8%
	26–35 years old	13	43.3%	9	30.0%	4	13.3%	12	40.0%	38	31.7%
	36–45 years old	3	10.0%	6	20.0%	1	3.3%	11	36.7%	21	17.5%
	46–55 years old	2	6.7%	0	0%	1	3.3%	1	3.3%	4	3.3%
	56 years old and above	2	6.7%	4	13.3%	1	3.3%	1	3.3%	8	6.7%
	Total	30	100.0%	30	100.0%	30	100.0%	30	100.0%	120	100.0%

Source: Authors’ statistics.

The total questionnaire results for all four garden spaces showed that there were 54 males and 66 females; 81 from within Guangdong Province and 39 from outside Guangdong Province; 18 with education below high school level, 92 with bachelor’s degrees, and 10 with postgraduate degrees or above; and 49 aged 16–25, 38 aged 26–35, 21 aged 36–45, 4 aged 46–55, and 8 aged 56 and above. Both for the four gardens themselves and for the entire sample, the background distribution of the sample conformed to the frequency distribution after the questionnaire was randomly distributed, with no obvious extreme frequency distribution, and the proportion of each sample’s background source was relatively reasonable. Given the reasonable frequency distribution of the sample, the collected questionnaires were further coded with word pair evaluations to obtain scores based on questions 5 to 20, and these scores were then statistically and visually analyzed.

#### 3.1.2 Reliability and validity analysis

The questionnaire obtained was first subjected to reliability and validity analysis to ensure the reasonableness and effectiveness of the questionnaire results. The software used to analyze data was IBM SPSS Statistics 26. In reliability testing, Cronbach’s alpha coefficient of 0.772 was obtained through a reliability analysis of the questionnaire scores, indicating that the information collected via the questionnaire was highly reliable and that there were very few instances of random completion.

Meanwhile, the total statistics of the items revealed that, among all, only the Cronbach’s alpha coefficients of visual occlusion, spatial compactness, and cultural elements increased slightly after deletion. This shows that there are some disputes and score differences among these three items, possibly due to differing opinions among the respondents (Table 5).

**Table 5:** Overall item totals of questionnaire evaluation scale scores.

Item	Scaled mean after removing items	Scaled variance after removing items	Correlation between adjusted items and total	Cronbach’s alpha after removing items
Visual impression	23.20	90.464	0.392	0.759
Area size	23.81	90.240	0.224	0.775
Natural elements	22.88	91.320	0.389	0.760
View from garden entrance	23.93	83.683	0.514	0.746
View from building entrance	23.98	84.663	0.480	0.750
Satisfaction with heights	23.68	84.571	0.470	0.750
Sense of enclosure	23.41	92.429	0.196	0.774
Visual obstruction	23.79	94.133	0.119	0.781
Visual hierarchy	23.33	88.207	0.478	0.752
Spatial compactness	23.53	94.974	0.102	0.782
Cultural elements	25.23	91.840	0.163	0.781
Richness of visual content	23.48	86.789	0.598	0.745
Integrity of color tone	23.30	87.674	0.492	0.751
Color richness	23.81	85.316	0.490	0.749
Perception of light and shadow	23.56	85.123	0.588	0.743
Shading measures	23.48	88.420	0.426	0.756

Source: Authors’ statistics.

#### 3.1.3 The questionnaire’s structural validity

Statistical analysis of 120 questionnaires in this study using the KMO and Bartlett tests showed that the KMO value was 0.759, falling within the range of 0.7 to 0.8, and the Bartlett *p* value was 0.000, falling within the range of less than or equal to 0.01, both indicating suitability for factor analysis. Based on the combined KMO and Bartlett P tests, the survey sample was deemed to have passed the reliability and validity tests for factor analysis, indicating that the information collected with the questionnaire design was generally reasonable, and was therefore suitable for implementation (Table 6).

**Table 6:** KMO and Cronbach’s test.

KMO sampling appropriateness measure	Inspection indicators	0.759
Bartlett’s sphericity test	Approximate chi-square	575.249
	Degrees of freedom	120
	Significance	0.000

Source: Authors’ statistics.

**Figure 6:**
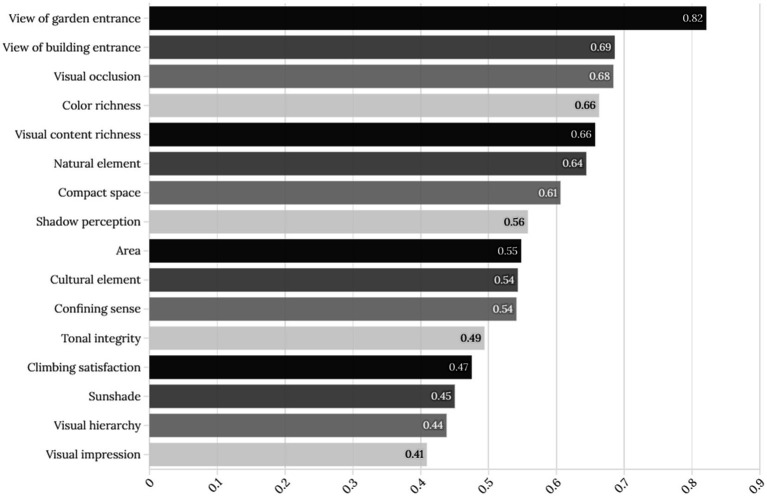
Common factor ANOVA.

We conducted an exploratory factor analysis on 120 questionnaires using SPSS software (version 26.0.0.0). We extracted factors using principal component analysis and rotated them using the maximum variance rotation method. The extracted values of each indicator can be obtained by using factor analysis for dimensionality reduction to determine the common factor variance. These extracted values represent the degree of explanation for each indicator in the principal component extraction. The most cited explanation is the view at the garden entrance, followed by the view at the building entrance, natural elements, visual obstruction, color richness, and visual content richness (Table 7; Figure 6).

**Table 7:** Dimensionality reduction factor analysis: common factor variance.

No.	Indicator name	Extraction results
1	Visual impression	0.409
2	Area size	0.548
3	Natural elements	0.644
4	View from garden entrance	0.821
5	View from building entrance	0.686
6	Satisfaction with heights	0.475
7	Sense of enclosure	0.541
8	Visual obstruction	0.684
9	Visual hierarchy	0.438
10	Spatial compactness	0.606
11	Cultural elements	0.543
12	Richness of visual content	0.657
13	Integrity of color tone	0.494
14	Color richness	0.663
15	Perception of light and shadow	0.558
16	Shading measures	0.450

Source: Authors’ statistics.

Table 8 shows the total variance explained by the exploratory factor analysis. The results indicate that, based on the eigenvalue greater than 1 criterion, four common factors were extracted. The total initial eigenvalues of these four factors are 4.433 + 1.931 + 1.519 + 1.336 = 9.219, explaining a cumulative variance of 57.617%. This indicates that these four common factors can explain more than half of the variance variation of the original variables, demonstrating high information extraction efficiency. Therefore, the extracted common factors can be considered sufficiently representative of the original indicators. The result also indicates that there is no common method bias in the questionnaire.

**Table 8:** Explanation of total variance of questionnaire indicator scores.

Element	Initial eigenvalues			Extracted sum of squares of loadings			Rotated Sum of squares of loadings		
	Total	Percentage of variance	Cumulative (%)	Total	Percentage of variance	Cumulative (%)	Total	Percentage of variance	Cumulative (%)
1	4.433	27.705	27.705	4.433	27.705	27.705	2.561	16.004	16.004
2	1.931	12.068	39.774	1.931	12.068	39.774	2.430	15.187	31.191
3	1.519	9.493	49.267	1.519	9.493	49.267	2.286	14.290	45.481
4	1.336	8.350	57.617	1.336	8.350	57.617	1.942	12.136	57.617
5	0.952	5.950	63.567						
6	0.827	5.169	68.736						
7	0.792	4.953	73.689						
8	0.742	4.634	78.323						
9	0.680	4.251	82.574						
10	0.622	3.888	86.463						
11	0.487	3.044	89.507						
12	0.417	2.604	92.111						
13	0.392	2.452	94.562						
14	0.362	2.260	96.822						
15	0.301	1.884	98.706						
16	0.207	1.294	100.000						

Source: Authors’ statistics.

Subsequently, the scores of each indicator in the four principal components were visualized and analyzed, and the indicator factors with obvious clusters were classified. The component score coefficient matrix shows the score coefficient of each indicator in the four components. The score coefficient fluctuates between −0.29 and 0.43. A high score coefficient indicates that the indicator has a high explanatory power and strong representativeness in the target component; conversely, a low score indicates that the indicator has a low explanatory power and lacks representativeness in the target component (Table 9).

**Table 9:** Component score coefficient matrix.

Indicator	Element			
	1	2	3	4
Visual impression	0.241	−0.099	0.066	−0.041
Area size	−0.160	0.386	−0.057	−0.133
Natural elements	0.400	−0.125	−0.087	−0.042
View from garden entrance	0.034	−0.130	0.406	−0.087
View from building entrance	0.022	−0.112	0.374	−0.060
Satisfaction with heights	−0.033	0.008	0.275	0.051
Sense of enclosure	−0.118	0.030	0.106	0.359
Visual obstruction	−0.007	0.007	−0.050	0.427
Visual hierarchy	0.215	0.017	−0.011	0.019
Spatial compactness	0.244	−0.144	−0.152	0.328
Cultural elements	−0.287	0.064	0.310	0.198
Richness of visual content	0.004	0.297	−0.065	0.141
Integrity of color tone	0.187	0.119	−0.077	−0.009
Color richness	−0.097	0.381	−0.034	−0.007
Perception of light and shadow	0.207	0.029	0.021	0.061
Shading measures	0.006	0.272	−0.067	0.001

Source: Authors’ statistics.

**Figure 7:**
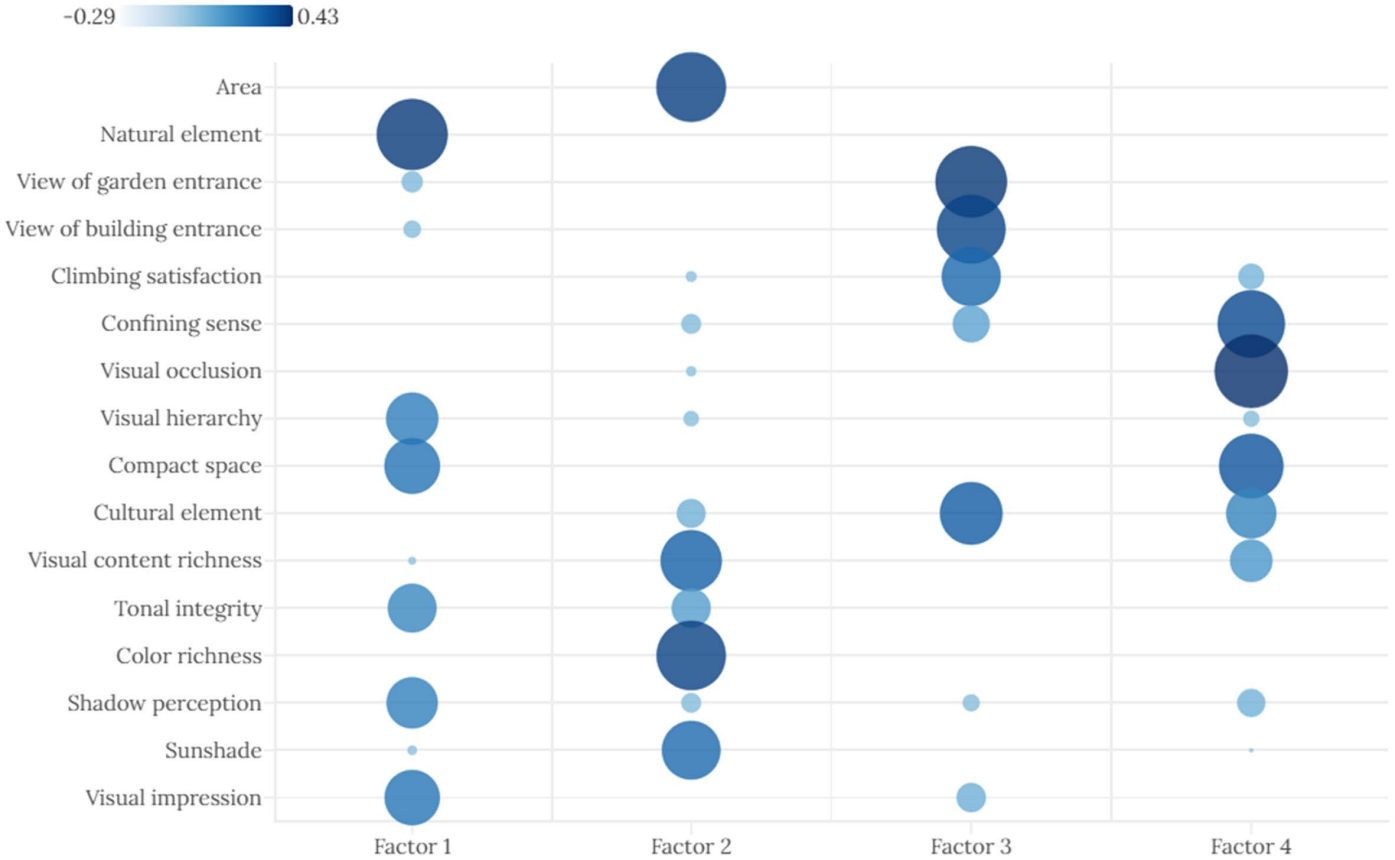
Component score coefficient matrix dot plot.

The resulting dot plot of the score coefficient matrix can express the clustering relationship between the indicators and components through the use of the index names in the component score coefficient matrix table as the Y-axis, the component classification as the X-axis, and the score coefficients as the radii of the points (Figure 7). Component 1 includes indicators related to visual semantics, such as the natural elements, visual impressions, overall color scheme, and distinct visual hierarchy, tending towards an expression of holistic attention to visual objects; therefore, Component 1 is classified as a “visual attention factor.” Component 2 includes indicators related to visual information, such as area size, richness of visual content, and richness of color, tending towards an expression of visual content; therefore, Component 2 is classified as a “visual content factor.” Component 3 includes indicators related to sightlines, such as satisfaction from elevation, views from garden entrances, and views from building entrances, tending towards an expression of the visual field; therefore, Component 3 is classified as a “visual field factor.” Component 4 includes indicators related to spatial enclosure and spatial compactness in the spatial experience process, primarily expressing the visual perception state within a spatial environment; therefore, Component 4 is classified as a “spatial visual order factor”.

In summary, principal component factor analysis of the score samples for each indicator reveals that the four most influential aspects of visual cognition are visual attention factors, visual information content factors, visual field factors, and spatial visual factors. This conclusion provides a dimensional reference for studying the laws governing visual cognition in the external space of Lingnan garden architecture and provides objective data to correct subjective cognitive experiences. In short, it comprehensively answers the question of where people should first look in this space (visual attention), then what they should look at (visual content), then how they should look (visual field and line of sight). Finally, a spatial cognitive concept is formed in their minds.

#### 3.1.4 Evaluation factor statistics

**Figure 8:**
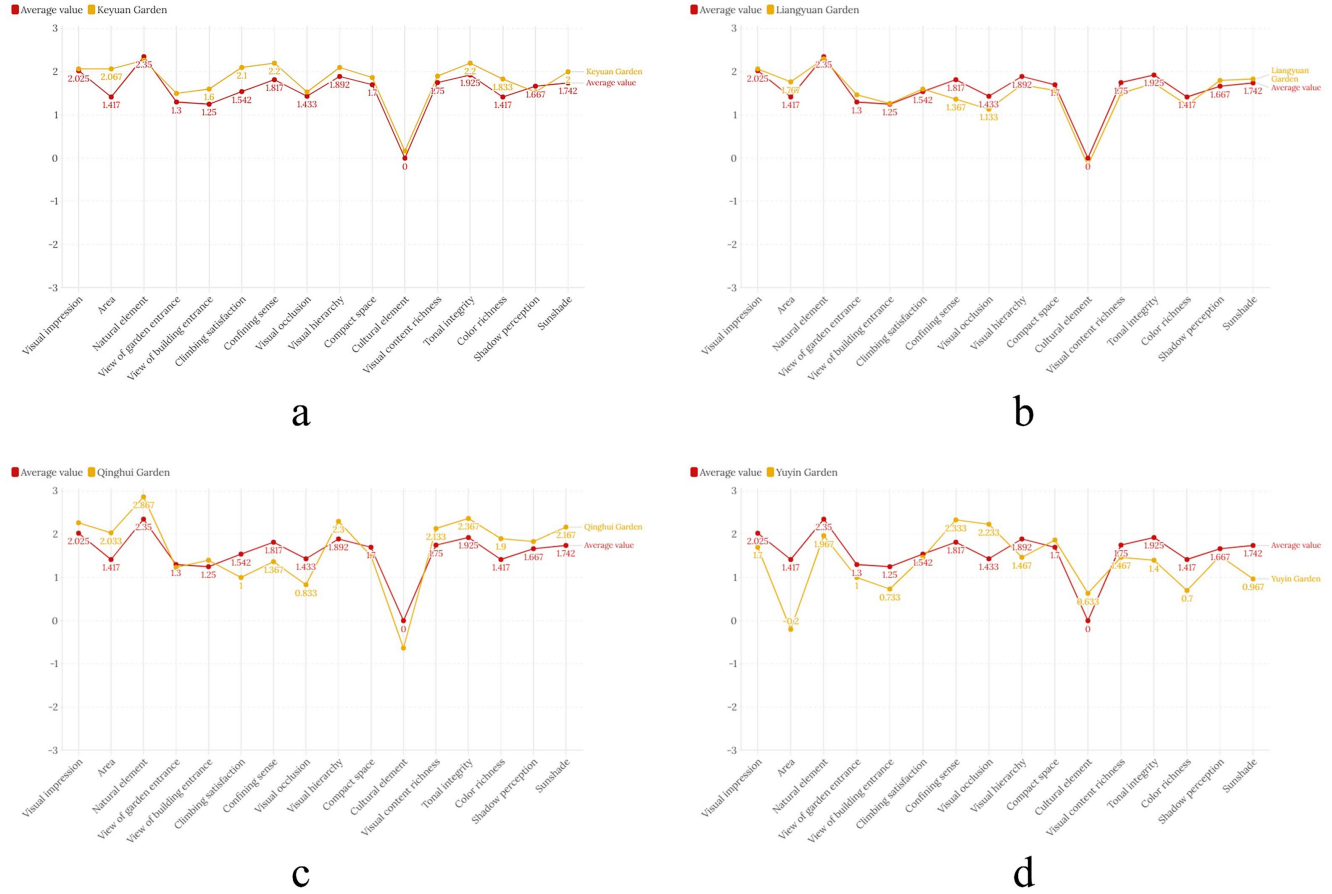
The average scores of the four Lingnan gardens compared with the overall score of all samples. **(a)** Keyuan Garden SD semantic scale broken line. **(b)** Liangyuan Garden SD semantic scale broken line. **(c)** Qinghui Garden SD semantic scale broken line. **(d)** Yuyin Garden SD semantic scale broken line.

Descriptive statistical analysis was performed on the sample scores of the questionnaires from the four gardens to measure the scores of each evaluation factor. Figure 8 presents the average scores of the four Lingnan gardens and compares them with the overall score of all samples.

##### 3.1.4.1 Keyuan Garden

The descriptive statistics of the evaluation factors for Keyuan Garden showed that the overall evaluations of the respondents tended to be uniform (Table 10). Looking at the maximum and minimum values, only the building entrance view and cultural elements showed two completely opposite scores among the evaluation factors; the scores of the other evaluation factors fluctuated within a certain range, reflecting that respondents had different opinions on the questions “Is the view from the building entrance open?” and “How much did you perceive the European classic elements?” in Keyuan Garden. From the perspective of variance, the spatial compactness and cultural elements showed larger variances, reflecting significant differences in respondents’ evaluations of these two issues and numerous inconsistencies in their scores. The average score of Keyuan Garden was expressed as a line graph of the SD scale and compared with the average scores of the four gardens. It can be found that Keyuan Garden’s overall score is close to the average score (Figure 8a).

**Table 10:** Descriptive statistics of various evaluation factors of Keyuan Garden.

No.	Item	Minimum value	Maximum value	Mean	Standard deviation	Variance
1	Visual impression	1	3	2.07	0.640	0.409
2	Area size	1	3	2.07	0.640	0.409
3	Natural elements	0	3	2.27	0.828	0.685
4	View from garden entrance	−1	3	1.50	1.106	1.224
5	View from building entrance	−3	3	1.60	1.192	1.421
6	Satisfaction with heights	−1	3	2.10	1.029	1.059
7	Sense of enclosure	0	3	2.20	0.761	0.579
8	Visual obstruction	−1	3	1.53	1.137	1.292
9	Visual hierarchy	1	3	2.10	0.803	0.645
10	Spatial compactness	−2	3	1.87	1.432	2.051
11	Cultural elements	−3	3	0.17	1.533	2.351
12	Richness of visual content	0	3	1.90	0.607	0.369
13	Integrity of color tone	−1	3	2.20	0.925	0.855
14	Color richness	−1	3	1.83	0.950	0.902
15	Perception of light and shadow	−1	3	1.53	1.196	1.430
16	Shading measures	0	3	2.00	0.947	0.897

Source: Authors’ statistics.

##### 3.1.4.2 Liangyuan Garden

Similarly, the descriptive statistics of the evaluation factors for Liangyuan Garden show that the overall evaluations of the participants tend to be uniform (Table 11). Looking at the maximum and minimum values, only the sense of enclosure and cultural elements showed two completely opposite scores among the evaluation factors; the scores of the other evaluation factors fluctuated within a certain range, reflecting different opinions among the participants regarding the questions “How much of a sense of enclosure did you feel in the courtyard?” and “How much of a sense of Western elements did you feel?” The variances for the garden entrance view, sense of enclosure, and cultural elements were relatively large, indicating significant differences in the participants’ evaluations of these three issues and numerous instances of inconsistent perceptions. The average score of Liangyuan Garden was expressed as a line graph of the SD scale and compared with the average scores of the four gardens. It can be found that Liangyuan Garden’s overall score is close to the average score (Figure 8b).

**Table 11:** Descriptive statistics of various evaluation factors of Liangyuan Garden.

No.	Item	Minimum value	Maximum value	Mean	Standard deviation	Variance
1	Visual impression	−1	3	2.07	1.015	1.030
2	Area size	−2	3	1.77	1.135	1.289
3	Natural elements	−1	3	2.30	0.952	0.907
4	View from garden entrance	−2	3	1.47	1.456	2.120
5	View from building entrance	−2	3	1.27	1.258	1.582
6	Satisfaction with heights	−1	3	1.60	1.163	1.352
7	Sense of enclosure	−3	3	1.37	1.450	2.102
8	Visual obstruction	−2	3	1.13	1.358	1.844
9	Visual hierarchy	−1	3	1.70	1.343	1.803
10	Spatial compactness	−2	3	1.57	1.251	1.564
11	Cultural elements	−3	3	−0.17	1.621	2.626
12	Richness of visual content	−1	3	1.50	1.137	1.293
13	Integrity of color tone	−1	3	1.73	1.081	1.168
14	Color richness	−1	3	1.23	1.040	1.082
15	Perception of light and shadow	−2	3	1.80	1.349	1.821
16	Shading measures	−1	3	1.83	1.262	1.592

Source: Authors’ statistics.

##### 3.1.4.3 Qinghui Garden

The descriptive statistics of the evaluation factors for Qinghui Garden show that there is a relatively large degree of inconsistency in the overall evaluations of the participants compared to other garden survey samples (Table 12). Looking at the maximum and minimum values, two completely opposite scores appeared for the evaluation factors of garden entrance view, building entrance view, satisfaction from elevation, sense of enclosure, visual obstruction, spatial compactness, and cultural elements. The scores for the remaining evaluation factors fluctuated within a certain range. This reflects that participants in Qinghui Garden have different opinions on questions such as “Is the view from the garden entrance open?”, “Is the view from the building entrance open?”, “Does it satisfy the desire to go to a higher point in the garden to see the whole view?”, “How much enclosure do participants feel in the courtyard?”, “Is there a lot of visual obstruction in the garden?”, “Do they feel a significant sense of spatial compactness?”, and “How much do they perceive European classic elements?” The variance of the above evaluation factors is relatively large, reflecting significant differences in participants’ evaluations on these issues and a high degree of inconsistency in their perceptions.

**Table 12:** Descriptive statistics of various evaluation factors of Qinghui Garden.

No.	Item	Minimum value	Maximum value	Mean	Standard deviation	Variance
1	Visual impression	0	3	2.27	0.828	0.685
2	Area size	−1	3	2.03	1.129	1.275
3	Natural elements	2	3	2.87	0.346	0.120
4	View from garden entrance	−3	3	1.23	1.794	3.220
5	View from building entrance	−3	3	1.40	1.734	3.007
6	Satisfaction with heights	−3	3	1.00	1.894	3.586
7	Sense of enclosure	−3	3	1.37	1.903	3.620
8	Visual obstruction	−3	3	0.83	1.783	3.178
9	Visual hierarchy	−1	3	2.30	1.022	1.045
10	Spatial compactness	−3	3	1.50	1.676	2.810
11	Cultural elements	−3	2	−0.63	1.564	2.447
12	Richness of visual content	−1	3	2.13	0.937	0.878
13	Integrity of color tone	0	3	2.37	0.809	0.654
14	Color richness	−2	3	1.90	1.094	1.197
15	Perception of light and shadow	−1	3	1.83	1.147	1.316
16	Shading measures	−3	3	2.17	1.117	1.247

Source: Authors’ statistics.

Figure 8c shows the semantic differential (SD) scale comparison of Qinghui Garden and the average of the four Lingnan gardens. Qinghui Garden exhibits notable strengths in dimensions such as natural elements (score is 2.867), tonal integrity (2.367), and sunshade (2.167), where its scores significantly exceed the cross-garden average. In contrast, Qinghui Garden scores relatively low in cultural elements (−0.63) and visual occlusion (0.833), indicating potential areas for improvement when compared to the average performance of the four gardens. The differences between Qinghui Garden and the cross-garden average are minimal for dimensions like visual impression (Qinghui Garden: 2.27, Average: 2.025) and perception of light and shadow (Qinghui Garden: 1.83, Average: 1.667), suggesting alignment with typical Lingnan Garden perceptual characteristics in these aspects.

##### 3.1.4.4 Yuyin Garden

The descriptive statistics of the evaluation factors for Yuyin Garden reveal significant differences in the overall evaluations from the participants (Table 13). Looking at the maximum and minimum values, two diametrically opposed scores appeared for the evaluation factors of visual impression, area size, view from the garden entrance, view from the building entrance, satisfaction from elevation, cultural elements, and richness of visual content. The scores for the remaining evaluation factors fluctuated within a certain range, reflecting differing opinions among the participants regarding the relevant issues at Yuyin Garden. The variance of the evaluation factors is relatively large, indicating significant differences in the participants’ evaluations of these three issues and numerous instances of inconsistent perceptions.

**Table 13:** Descriptive statistics of various evaluation factors of Yuyin Garden.

No.	Item	Minimum value	Maximum value	Mean	Standard deviation	Variance
1	Visual impression	−3	3	1.70	1.512	2.286
2	Area size	−3	3	−0.20	1.901	3.614
3	Natural elements	−1	3	1.97	1.299	1.689
4	View from garden entrance	−3	3	1.00	1.390	1.931
5	View from building entrance	−3	2	0.73	1.437	2.064
6	Satisfaction with heights	−3	3	1.47	1.502	2.257
7	Sense of enclosure	1	3	2.33	0.711	0.506
8	Visual obstruction	−1	3	2.23	0.935	0.875
9	Visual hierarchy	−2	3	1.47	1.074	1.154
10	Spatial compactness	0	3	1.87	0.937	0.878
11	Cultural elements	−3	3	0.63	1.520	2.309
12	Richness of visual content	−2	3	1.47	1.252	1.568
13	Integrity of color tone	−2	3	1.40	1.429	2.041
14	Color richness	−3	3	0.70	1.841	3.390
15	Perception of light and shadow	−1	3	1.50	1.075	1.155
16	Shading measures	−2	3	0.97	1.129	1.275

Source: Authors’ statistics.

Figure 8d shows the semantic differential (SD) scale comparison of Yuyin Garden and the average of the four Lingnan gardens. Yuyin Garden scored significantly higher than the mean in dimensions such as confining sense (Yuyin Garden: 2.333; average: 1.817) and visual occlusion (Yuyin Garden: 2.233; average: 1.433), which is closely related to its compact spatial layout of “a small mountain forest” (the mountain occupies five hundred square meters with the peak only seven meters high). Through the layering of buildings and vegetation, it enhances the sense of enclosure and the richness of visual hierarchy, demonstrating the spatial creation of a small, exquisite garden. The scores in dimensions such as area (Yuyin Garden: −0.2; average: 1.417) and view from building entrance (Yuyin Garden: 0.733; average: 1.25) are significantly lower than the average, reflecting the limitations of its spatial scale (as a small private garden, its area is inherently limited) and the lack of vertical landscape (such as the creation of climbing plants), which are characteristic shortcomings caused by its spatial type.

##### 3.1.4.5 Comparison results of four gardens

A comprehensive descriptive statistical analysis of the evaluation factors obtained from the field surveys of the four gardens reveals that, among the 120 questionnaires, the two factors with the highest variance were area size and cultural elements (Table 14), indicating significant differences in evaluation of these two factors and difficulty in reaching a consensus among the sample. Although the statistical results of the SD method are influenced by factors such as the participants’ educational level and background, the comprehensive statistical results also suggest, to some extent, that there is disagreement regarding the public’s visual perception of the external space of Lingnan garden architecture in terms of its smaller size and the prevalence of European classical cultural symbols. In other words, while area size and European classical cultural elements may be visual characteristics of the external space of Lingnan garden architecture in a qualitative description of visual perception, they are certainly not prominent factors in the mainstream public’s visual perception. Secondly, the factors with the largest variance are the garden entrance view, building entrance view, susceptibility to elevation, and visual obstruction. This indicates that people have certain differences in their perception of qualitative descriptions such as “climb high and gaze far” and “open and transparent, with a view of the mountains right from the door”.

**Table 14:** Descriptive statistics of various evaluation factors of the 4 gardens.

No.	Item	Minimum value	Maximum value	Mean	Standard deviation	Variance
1	Visual impression	−3	3	2.03	1.057	1.117
2	Area size	−3	3	1.42	1.580	2.497
3	Natural elements	−1	3	2.35	0.967	0.935
4	View from garden entrance	−3	3	1.30	1.453	2.111
5	View from building entrance	−3	3	1.25	1.439	2.071
6	Satisfaction with heights	−3	3	1.54	1.472	2.166
7	Sense of enclosure	−3	3	1.82	1.366	1.865
8	Visual obstruction	−3	3	1.43	1.424	2.029
9	Visual hierarchy	−2	3	1.89	1.114	1.240
10	Spatial compactness	−3	3	1.70	1.345	1.808
11	Cultural elements	−3	3	0.00	1.609	2.588
12	Richness of visual content	−2	3	1.75	1.039	1.080
13	Integrity of color tone	−2	3	1.93	1.139	1.297
14	Color richness	−3	3	1.42	1.357	1.842
15	Perception of light and shadow	−2	3	1.67	1.191	1.417
16	Shading measures	−3	3	1.74	1.199	1.437

Source: Authors’ statistics.

**Figure 9:**
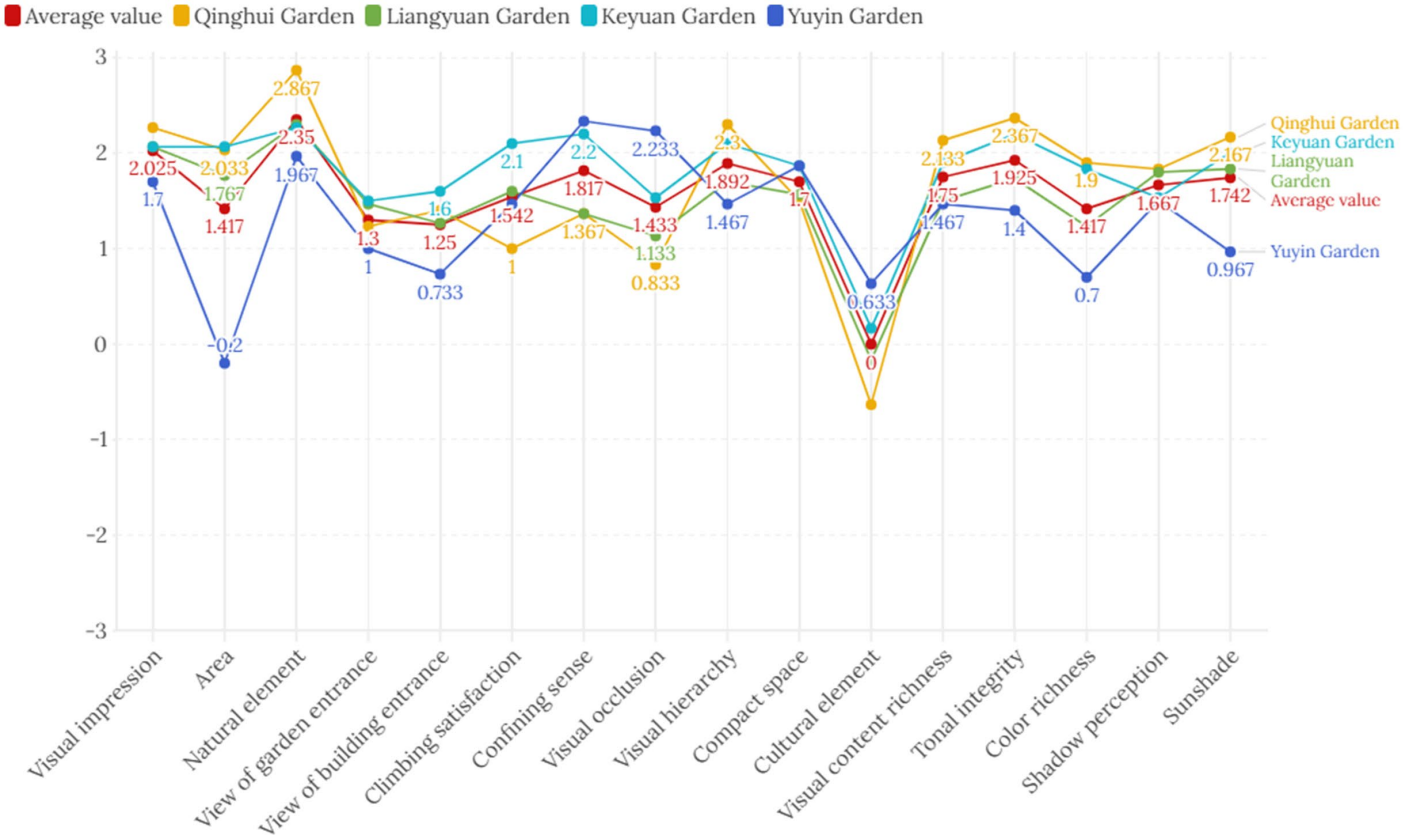
Linear graph of the SD semantic scale between the four major gardens of Lingnan and the overall average.

Descriptive statistical analysis of the questionnaires reveals discrepancies between people’s visual perception of external spaces and their experiential qualitative descriptions. Quantification is needed at the level of visual feature perception to compare the common patterns and viewpoints of the general public regarding the visual characteristics of Lingnan gardens’ external spaces. This would provide a direct reflection of the public’s level of acceptance of various evaluation criteria. The visualization of the statistical results allows for a more intuitive comparison of the relationships between the evaluation scales of each factor and the relationships between each garden evaluation factor and the overall population (Figure 9).

Figure 9 shows that, in the scale analysis of 120 questionnaires using semantic analysis, the overall trend of the visual evaluation factor scores for the external spaces of the four major Lingnan gardens is basically consistent and can serve as a dimension for studying the patterns of visual cognitive characteristics of the external spaces of Lingnan garden architecture. Individual gardens exhibit some unique characteristics while maintaining overall commonalities. For example, Qinghui Garden had the highest average score for natural elements, indicating that respondents had the clearest perception of natural elements such as vegetation in Qinghui Garden’s visual perception. Yuyin Garden had the lowest average score for perceived size, indicating that respondents generally perceived it as having a smaller spatial size. Given the common perception that Western culture has a lower proportion of garden spaces, Qinghui Garden had the lowest average score, indicating that respondents had the highest perception of Chinese elements in the external spaces of Qinghui Garden’s architecture. The evaluation factor scores for Yuyin Garden fluctuated the most compared to other garden spaces.

### 3.2 Differences and preferences among factors

To explore the intergroup differences between four common visual perception factors and four demographic variables, a total of 16 independent statistical hypothesis tests were conducted in this study. However, such a multiple comparison scenario would lead to the inflation of the family-wise error rate, meaning that with the increase of test times, the probability of rejecting at least one true null hypothesis (Type I error) would far exceed the preset significance level (e.g., *α* = 0.05). Without statistical control for this issue, the reported “significant” differences are likely to be mixed with a considerable proportion of false discoveries, which would seriously weaken the internal validity and repeatability of the research conclusions.

To strike a prudent balance between statistical detection power and error control, the false discovery rate (FDR) correction procedure was adopted in this study. Unlike the traditional family-wise error rate control, the FDR method regulates the expected proportion of false rejections (i.e., false positives) among all rejected null hypotheses. Compared with the overly conservative Bonferroni correction, the FDR method significantly improves the statistical test power while constraining the overall proportion of false positives, which is particularly suitable for such exploratory and multi-dimensional perception research.

Based on the conclusions of principal component analysis (PCA), the four perception dimensions were taken as the objects of intergroup difference analysis. Specifically, the factor score of each subject was calculated first, followed by intergroup comparison at the factor score level, yielding 16 *p*-values corresponding to 4 groups and 4-dimension factors. These p-values were then sorted in ascending order, and the FDR critical value for each p-value was calculated according to the formula (rank/total number of tests) × 0.05. The position of the first p-value less than the corresponding FDR critical value was identified, and all p-values above this position were regarded as statistically significant. As shown in Table 15, after FDR correction, only the visual attention factor showed significant differences across age and education level groups.

**Table 15:** FDR correction for different groups and significance in four dimensions.

No.	Group	Principal component factor	F	*P*	FDR critical value
1	Age group	Visual attention factor	4.677	0.002*	0.003125
2	Education level	Visual attention factor	4.505	0.005*	0.00625
3	In Guangdong Province or outside Guangdong Province	Visual attention factor	1.75	0.024	0.009375
4	In Guangdong Province or outside Guangdong Province	Visual field factor	1.409	0.056	0.0125
5	Education level	Spatial visual order factor	2.605	0.078	0.015625
6	Age group	Visual field factor	1.916	0.112	0.01875
7	In Guangdong Province or outside Guangdong Province	Visual content factor	0.325	0.136	0.021875
8	Education level	Visual content factor	1.272	0.284	0.025
9	Gender group	Visual content factor	0.816	0.303	0.028125
10	Education level	Visual field factor	1.12	0.33	0.03125
11	In Guangdong Province or outside Guangdong Province	Spatial visual order factor	1.363	0.43	0.034375
12	Gender group	Spatial visual order factor	12.07	0.575	0.0375
13	Gender group	Visual field factor	1.584	0.623	0.040625
14	Age group	Visual content factor	0.421	0.793	0.04375
15	Gender group	Visual attention factor	2.87	0.857	0.046875
16	Age group	Spatial visual order factor	0.206	0.935	0.05

Source: Authors’ statistics. The corrected significance is determined by the relationship between the FDR critical value and the *P* value. If P< FDR critical value, it is considered that there is a significant difference in the sample. One asterisk (*): indicates that the result is significant, meaning *P* < 0.05, that is, the difference is statistically significant at a 95% confidence level.

## 4 Discussion

The semantic differential (SD) method was used in this study to systematically explore the public’s visual perception preferences for Lingnan garden spaces. Statistical tests showed that the overall reliability of the questionnaire was good (Cronbach’s α = 0.772), and the construct validity passed the KMO and Bartlett tests (KMO = 0.759, *p* < 0.0005), indicating that the data was suitable for factor analysis, which ensured the reliability of the research findings from a data perspective. However, at the specific factor level, there were significant differences in the scores of “visual occlusion,” “spatial compactness,” and “cultural elements,” and the reliability coefficients increased after their removal. This suggests that there are significant differences in public perception of these dimensions and reflects that the SD method may be greatly affected by individual experience differences when capturing complex spatial and cultural attributes. The result also highlights the inherent characteristics of subjective assessment methods—while they can effectively capture collective perception trends, they cannot avoid the “fluctuations” brought about by individual cognitive backgrounds. This study revealed the misalignment between the public’s cognitive structure and objective spatial characteristics through subjective perception data, in contrast to previous studies on Lingnan gardens that mostly relied on objective mapping, climate data, or style classification. For example, “size” and “cultural elements” were the two areas with the greatest disagreement in evaluation, which contrasts with the objective descriptions in the past literature that often emphasized the “small and exquisite” characteristics and “fusion of Chinese and Western styles” of Lingnan gardens. This work illustrates, on the one hand, that subjective assessments can reveal cognitive truths obscured by traditional objective descriptions—the public may not necessarily regard the “size” or “cultural symbols” that scholars focus on as the core of their visual experience. On the other hand, it also warns us that relying solely on subjective reports may not accurately reflect the objective state of spatial physical attributes; only by combining both can a more complete cognitive picture be formed.

Further analysis of the collected samples revealed that visual perception exhibits a clear four-dimensional structure (visual attention, visual information, visual field, and spatial visual order), with a cumulative explanatory power of 57.61%. This echoes yet differs from factors such as “naturalness” and “sense of shelter” extracted in Zhao et al.'s (2020) study on spatial perception in urban forests. The unique “visual information factors” (including color, content richness, etc.) of Lingnan gardens highlight their decorative and detailed regional characteristics.

Descriptive statistics indicate that the public has a high degree of consensus on the visual perception of Lingnan gardens in terms of natural elements, spatial hierarchy, tonal integrity, and shading design. These dimensions received stable and above-average scores, confirming the core feature of Lingnan gardens as a harmonious system of nature and human intervention (Gobster et al., 2007; Ulrich, 1981). Among them, the outstanding performance of natural elements is highly consistent with the traditional garden-making tradition of Lingnan gardens that emphasizes the integration of plants, water features, and rocks (Zheng, 2019); while the high scores for shading measures and color integrity support the adaptive design strategies for the humid and hot climate from the perception level (Liang and Li, 2019; Xue and Xiao, 2016).

However, in the two dimensions of cultural elements (Chinese-Western fusion) and area size, public evaluations show significant differences. This contrasts with the feature often emphasized by the academic community, “Chinese-Western integration” (Zhang W. et al., 2024; Zhong, 2020), suggesting that such symbols may not be prominent in visual presentation or public interpretation. This finding is consistent with the results of factor analysis, that is, cultural elements and area do not constitute independent perception dimensions, reflecting the potential deviation between objective characteristics and subjective cognition.

The results of the chi-square test suggest that within the sample range of this study, the inter-group difference patterns of perception evaluation are relatively concentrated. Specifically, statistically significant correlations mainly occur in the “visual attention” dimension and are only related to the two variables of age and educational level. Compared with static attributes such as gender and geographical origin, age and educational level are dynamic “growth factors,” usually associated with the richness of personal experience, the construction of knowledge systems, and the development of aesthetic cognition. Therefore, this finding of this study may suggest that the growth and changes of individuals in these aspects might have a potential impact on their distribution pattern of visual attention and focus of attention in the Lingnan garden environment.

On the other hand, in the three dimensions of “visual content,” “field of view” and “spatial visual order”, this analysis did not detect significant group differences. This might indicate that the public’s perception and evaluation of basic visual attributes such as color, material, visual transparency, and layout logic in gardens show a higher degree of consistency and are relatively less influenced by individual background characteristics.

Based on the above findings, we can initially speculate that in the visual experience of Lingnan gardens, the “visual attention” dimension involving cognitive integration and meaning interpretation may be more sensitive to the growth background of visitors. The perception related to basic visual attributes may have a stronger universality. If this speculation can be confirmed by subsequent research, it can provide a reference for the refined interpretation of gardens and public education.

It should be emphasized that the above interpretation is derived from the specific samples of this exploratory study. The robustness and universality of the group difference model, especially the independence of the effects of age and educational level, still need to be tested through larger-scale, more systematic stratified samples and statistical models that can control confounding variables.

This study has certain limitations. Firstly, the data collection period was relatively limited, with the survey completed within 3 weeks. This failed to incorporate seasonal variations, dynamic differences in weather conditions, and the evolution of tourist group perception preferences over time. It’s important to note that in this exploratory study, our primary objective was to identify the basic structure of visual perception, rather than measuring absolute, seasonally independent perceptual scores. We chose to conduct the survey in early November, a period when Guangdong, located in the subtropical Lingnan region, experiences a mild climate, evergreen vegetation, and a stable visual environment—ideal for garden visits and also the peak tourist season. This helps control extreme seasonal variables, such as summer heat, typhoons, or winter cold fronts, which can significantly alter sensory experiences and tourist composition. Furthermore, to minimize intraday and intraweekly biases during the survey period, we distributed questionnaires on multiple dates, including weekdays and weekends, to reduce the interference of time-of-day differences with sample representativeness. Nevertheless, the subtle differences in vegetation and weather conditions across the four seasons in the Lingnan region were not considered. The tourist sample covered by the short-term survey cannot fully reflect the visual perception preferences of tourist groups at different times, which to some extent affects the stability and external validity of the research results. Furthermore, longer-term, cross-seasonal studies would undoubtedly provide richer data on perceptual stability. Future research could extend the survey period to the whole year, conducting semantic difference surveys in batches according to seasons, incorporating control variables such as weather and time of day, and combining long-term tracking data to analyze the dynamic changes in visual perception preferences, further enhancing the generalizability of the research conclusions.

## 5 Conclusion

The visual perception characteristics and group differences of traditional Lingnan garden spaces were explored in this study through principal component analysis, descriptive statistical analysis, and Chi-square tests, and there were several findings about Lingnan gardens in visual perception based on the collected sample data and analysis. (1) Firstly, regarding the commonalities characteristics of Lingnan gardens in public perception, four main dimensions affecting public visual perception were identified via principal component factor analysis: visual attention factors, visual information content factors, field of vision factors, and spatial visual factors. (2) The overall trend of scores for all samples tends to be consistent, and the visual perception results of Lingnan gardens in different locations are similar, indicating that the method has satisfactory applicability to the garden system. Natural elements scored the highest, and the public’s evaluation of this issue was the most unified. At the same time, the evaluation of area size and Western cultural elements (mainly European elements) had the most significant differences, and it was difficult to unify the opinions of the respondents. (3) Among the perception dimensions, only the “visual attention factor” showed significant differences between the two groups in terms of age and educational level. The growth factors have a certain influence on the visual attention factor. There were no significant differences in the other dimensions and groups. (4) This research method is highly applicable to the study of Lingnan gardens and can also be applied to other gardens within the same garden system. However, compared to other gardens, the cultural scope of Guangdong is relatively clear, while the boundaries of Jiangnan gardens and Northern gardens are more blurred and difficult to define. At the same time, cultural level and age group are factors that significantly affect the results. Therefore, subsequent research needs to limit or clarify this variable to further verify the accuracy of this method.

In summary, this exploratory study applies the Semantic Differential method to propose a preliminary multi-dimensional framework for understanding visual perception in Lingnan garden spaces. Through factor analysis, it identifies four potential dimensions (visual attention, content, field, and spatial order) that may structure public cognition. The findings suggest a possible divergence between quantified public perceptions and certain traditional qualitative descriptions, particularly regarding garden scale and cultural elements. While common perceptual patterns were observed across the four gardens studied, some garden-specific characteristics were also noted. Furthermore, the analysis points to several demographic factors, most notably education level and ag, that appear to be associated with variations in specific perceptual dimensions.

These insights may offer a tentative empirical reference for future theoretical models of garden perception. On a practical level, they could inform considerations for heritage management and contemporary design by highlighting perceptual dimensions (e.g., natural elements, color integrity) that received high and consistent public appraisal. However, given the study’s methodological constraints, including its sample size and convenience sampling approach, these implications should be treated as suggestive rather than definitive.

Future research would benefit from employing larger, more stratified samples across multiple seasons and incorporating complementary methods (e.g., eye-tracking, interviews) to validate and extend the perceptual framework proposed here. Expanding the scope to include a wider variety of Lingnan garden types would also enhance the generalizability of findings.

## Data Availability

The original contributions presented in the study are included in the article/Supplementary material, further inquiries can be directed to the corresponding authors.
