# Supplementary material for: Visual perception preferences in Lingnan gardens: a semantic differential survey with a convenience sample
Source: Front Psychol. 2026 Mar 9;17:1750378. doi: 10.3389/fpsyg.2026.1750378 (PMC13006295; doi:10.3389/fpsyg.2026.1750378)
Supplement: Supplementary file 1 [file Supplementary_file_1.docx]

Appendix A: Questionnaire Survey on the Perception of Lingnan Garden Landscape

Please note that the information provided below is translated from the original questionnaire, which was written in Chinese.

Part I: Informed Consent

This survey will serve as a baseline for understanding satisfaction with the park environment. The survey targeted individuals aged 16 and above. We would appreciate it if you could take approximately 15 minutes to honestly and proactively share your perceptions or opinions on the landscapes of four traditional gardens in Lingnan. If you agree to participate in the study, please complete the following questionnaire. Please note that you may withdraw at any time. All information related to this survey will be kept anonymous and will not be used for any purpose other than academic research.

(1) Agree with the survey [Single Choice]

□ Agree

□ Disagree (Terminate survey)

(2) Have you visited traditional gardens in Lingnan (Yuyin Garden in Guangzhou, Foshan Liangyuan Garden, Dongguan Keyuan Garden, or Shunde Qinghui Garden)? [Single Choice]

□ Yes (Continue to the Table A1)

□ No (Terminate survey)

Thank you for your participation!

**Table A1.** Questionnaire survey on the perception of Lingnan garden landscape.

| Select the Lingnan gardens you have visited. | |  | □ Yuyin Garden in Guangzhou  □ Foshan Liangyuan Garden  □ Dongguan Keyuan Garden  □ Shunde Qinghui Garden | | | | | | | | |  |
| --- | --- | --- | --- | --- | --- | --- | --- | --- | --- | --- | --- | --- |
| **No.** | **Question** |  | **Scoring Questions** | | | | | | | | |  |
| 1 | What are your impressions of this garden space? | / | Profound | 3 | 2 | 1 | 0 | -1 | -2 | -3 | Simple | / |
| 2 | What is the size of the garden? | / | Large | 3 | 2 | 1 | 0 | -1 | -2 | -3 | Small | / |
| 3 | Are there many natural elements in the garden, such as plants, water features, and rocks? | 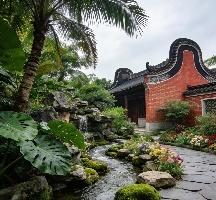 | Numerous | 3 | 2 | 1 | 0 | -1 | -2 | -3 | Few | 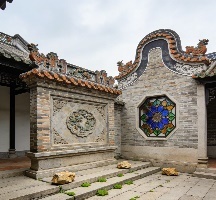 |
| 5 | Is the view from the garden entrance open? | 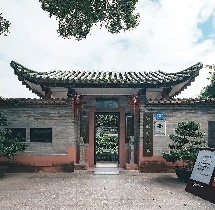 | Open | 3 | 2 | 1 | 0 | -1 | -2 | -3 | Limited | 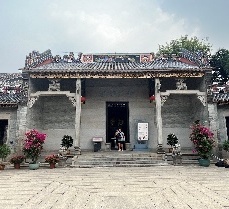 |
| 6 | Is the view from the building entrance open? | 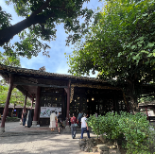 | Expansive | 3 | 2 | 1 | 0 | -1 | -2 | -3 | Limited | 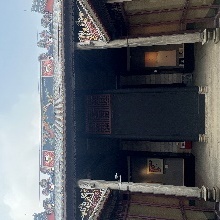 |
| 7 | How enclosed is the courtyard? | 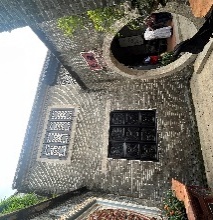 | Intense | 3 | 2 | 1 | 0 | -1 | -2 | -3 |  | 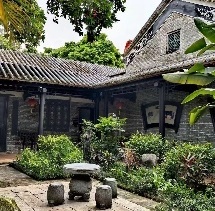 |
| 8 | Are there many visual obstructions in the garden? | 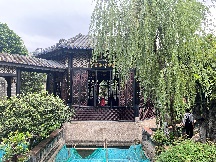 | Closed | 3 | 2 | 1 | 0 | -1 | -2 | -3 | Open | 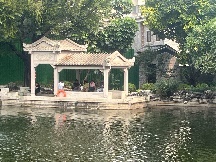 |
| 9 | Are the visual layers in the garden rich? | 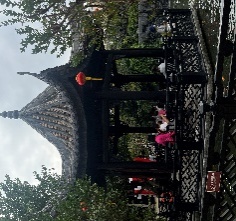 | Many | 3 | 2 | 1 | 0 | -1 | -2 | -3 | Few | 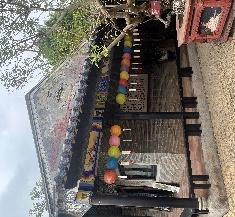 |
| 10 | Does the space feel noticeably cramped? | 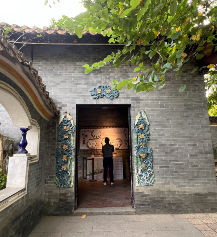 | Clear | 3 | 2 | 1 | 0 | -1 | -2 | -3 | Vague | 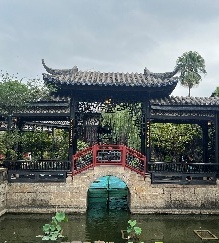 |
| 11 | How much of a Western style is present? | 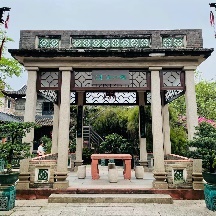 | Obvious | 3 | 2 | 1 | 0 | -1 | -2 | -3 | Vague | 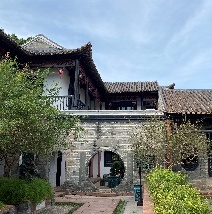 |
| 12 | How rich are the visual elements seen in the courtyard? | 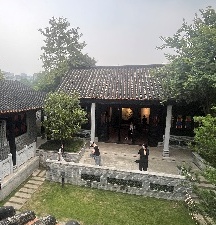 | Many | 3 | 2 | 1 | 0 | -1 | -2 | -3 | Few | 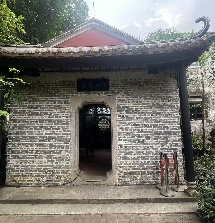 |
| 13 | Do you feel the overall color harmony of the garden? | 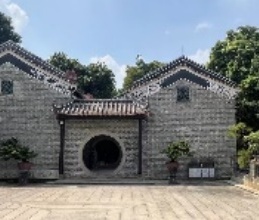 | Rich | 3 | 2 | 1 | 0 | -1 | -2 | -3 | Monotonous | 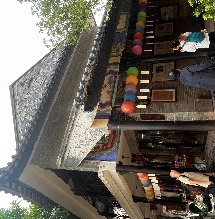 |
| 14 | How rich are the colors on the exterior of the buildings? | 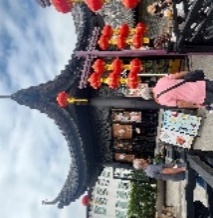 | Unified | 3 | 2 | 1 | 0 | -1 | -2 | -3 | Disorderly | 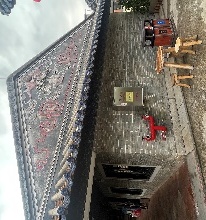 |
| 15 | How pronounced is the interplay of light and shadow in the garden? | 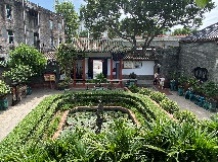 | Rich | 3 | 2 | 1 | 0 | -1 | -2 | -3 | Monotonous | 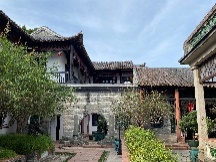 |
| 16 | Are the sunshade measures in the garden adequate? | 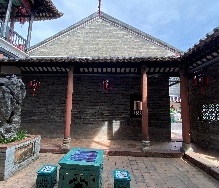 | Obvious | 3 | 2 | 1 | 0 | -1 | -2 | -3 | Vague | 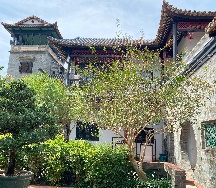 |

Source: authors’ statistics.

Part II: Demographic characteristics

(1) What is your gender? [Single Choice]

□ Male

□ Female

(2) From/Outside Guangdong Province? [Single Choice]

□ Within the province

□ Outside the province

(3) What is your current level of education? [Single Choice]

□ High school and below

□ University

□ Graduate and above

(4) Age Group [Single Choice]

□ 16-25 years old

□ 26-35 years old

□ 36-45 years old

□ 46-55 years old

□ 56 years old and above
